# Inhibition of NF-κB with an Analog of Withaferin-A Restores TDP-43 Homeostasis and Proteome Profiles in a Model of Sporadic ALS

**DOI:** 10.3390/biomedicines12051017

**Published:** 2024-05-05

**Authors:** Pooja Shree Mishra, Daniel Phaneuf, Hejer Boutej, Vincent Picher-Martel, Nicolas Dupre, Jasna Kriz, Jean-Pierre Julien

**Affiliations:** 1 CERVO Brain Research Centre, 2601 Chemin de la Canardière, Quebec, QC G1J 2G3, Canada; poojashri.mishra@gmail.com (P.S.M.); daniel.phaneuf.2@ulaval.ca (D.P.); hboutej1@gmail.com (H.B.); jasna.kriz@fmed.ulaval.ca (J.K.); 2 Division of Neurosciences, Centre Hospitalier Universitaire de Québec, Laval University, Quebec, QC G1V 4G2, Canada; vincent.picher-martel.1@ulaval.ca (V.P.-M.); nicolas.dupre@fmed.ulaval.ca (N.D.); 3 Department of Psychiatry and Neuroscience, Faculty of Medicine, Laval University, Quebec, QC G1V 0A6, Canada

**Keywords:** TDP-43, NF-κB, Withaferin-A, ALS, cerebrospinal fluid, proteome

## Abstract

The current knowledge on pathogenic mechanisms in amyotrophic lateral sclerosis (ALS) has widely been derived from studies with cell and animal models bearing ALS-linked genetic mutations. However, it remains unclear to what extent these disease models are of relevance to sporadic ALS. Few years ago, we reported that the cerebrospinal fluid (CSF) from sporadic ALS patients contains toxic factors for disease transmission in mice via chronic intracerebroventricular (i.c.v.) infusion. Thus a 14-day i.c.v. infusion of pooled CSF samples from ALS cases in mice provoked motor impairment as well as ALS-like pathological features. This offers a unique paradigm to test therapeutics in the context of sporadic ALS disease. Here, we tested a new Withaferin-A analog (IMS-088) inhibitor of NF-κB that was found recently to mitigate disease phenotypes in mouse models of familial disease expressing TDP-43 mutant. Our results show that oral intake of IMS-088 ameliorated motor performance of mice infused with ALS-CSF and it alleviated pathological changes including TDP-43 proteinopathy, neurofilament disorganization, and neuroinflammation. Moreover, CSF infusion experiments were carried out with transgenic mice having neuronal expression of tagged ribosomal protein (hNfL-RFP mice), which allowed immunoprecipitation of neuronal ribosomes for analysis by mass spectrometry of the translational peptide signatures. The results indicate that treatment with IMS-088 prevented many proteomic alterations associated with exposure to ALS-CSF involving pathways related to cytoskeletal changes, inflammation, metabolic dysfunction, mitochondria, UPS, and autophagy dysfunction. The effective disease-modifying effects of this drug in a mouse model based on i.c.v. infusion of ALS-CSF suggest that the NF-κB signaling pathway represents a compelling therapeutic target for sporadic ALS.

## 1. Introduction

ALS is a fatal neurodegenerative disease associated with motor neuron degeneration and muscular atrophy. The disease currently lacks effective treatments. Mutations in several genes have been implicated in familial ALS including SOD1, C9ORF72, FUS, UBQLN2, SQSTM1, ATXN2, OPTN, VCP, VAPB, DCTN1, and TARDBP encoding TAR DNA-binding protein 43 (TDP-43) [1]. Some of the genetic mutations linked to familial ALS have also been detected at low frequency in sporadic ALS (sALS) [1,2,3]. Yet, for the most part the etiology of sALS remains unknown. Most of the knowledge on ALS pathogenic mechanisms has been derived from studies with animal models bearing ALS-linked genetic mutations [4], but it remains unclear whether these disease models are of relevance to sporadic ALS (sALS).

A key pathological hallmark of both familial and sporadic ALS, which is also found in subsets of frontotemporal dementia (FTD) and Alzheimer’s disease, is the cytoplasmic accumulation and aggregation of TDP-43. There are reports suggesting that such TDP-43 proteinopathy may occur through self-seeding prion-like mechanisms and exosome transmission [5]. This concept of disease propagation is supported by the finding that human TDP-43 WT protein potentiated pathology when co-expressed with mutant TDP-43^Q331K^ in transgenic mice [6]. Few years ago, it was proposed that the cerebrospinal fluid (CSF) from ALS patients may contain toxic factors for the spreading of disease [7]. To test this hypothesis, our group has carried out chronic intracerebroventricular (i.c.v.) infusion of CSF samples from sporadic ALS patients or controls into mice expressing human TDP-43 WT transgene which do not develop pathological features [8]. A 14-day infusion of ALS-CSF in these mice provoked ALS-like pathological features [8]. Thus, the ALS-CSF infusion in human TDP-43 transgenic mice offers a unique paradigm for testing therapeutics aiming to mitigate pathology in the context of sporadic ALS disease.

Here, we have used the ALS-CSF-infused mice to investigate the effects of a novel semi-synthetic Withaferin-A derivative called IMS-088. Withaferin-A, an active steroid lactone derived from Withania somnifera, was found previously to confer therapeutic benefits in different mouse models expressing ALS-linked mutant SOD1 or mutant TDP-43 proteins [9,10,11]. Withaferin-A acts as an antagonist of nuclear factor kappa-B (NF-κB) essential modulator (NEMO) [12]. We recently reported that inhibition of NF-κB, either with a super-repressor Iκ-B construct or by Withaferin-A treatment, led to induction of autophagy with ensuing reduction of TDP-43 proteinopathy in the brain and spinal cord of transgenic mice expressing human TDP-43 mutants [10,13]. Furthermore, oral administration of the 4-O-methyl Withaferin-A analog (called IMS-088), which is better tolerated than Withaferin-A in mice, was found to mitigate TDP-43 pathology, neurofilament abnormalities, and neuroinflammation in transgenic mice expressing TDP-43 mutants [14]. The IMS-088 analog also alleviated ALS-associated phenotypes in transgenic mice expressing FUSR521G mutant [15]. Here, we report that oral intake of Withaferin-A analog IMS-088 ameliorated motor performance and pathological changes in hTDP-43^WT^ mice infused with ALS-CSF. Of particular interest is the finding that IMS-088 treatment mitigated TDP-43 proteinopathy induced by ALS-CSF infusion in hTDP-43^WT^ mice. Moreover, IMS-088 rescued in part the neurofilament disorganization and neuronal translational defects triggered by infusion of ALS-CSF.

## 2. Materials and Methods

### 2.1. CSF Sample Collection and Study Groups

We obtained informed consent and collected CSF samples from patients diagnosed with ALS (ALS-CSF; n = 10; mean age 58.3 years) as well as from age-matched control subjects with normal pressure hydrocephalus pathology (NALS-CSF, n = 5; mean age 67.6 years) [8]. Both, the ALS-CSF as well as the NALS-CSF samples were separately pooled for investigation.

We studied the effect of CSF in two different mouse models for their potential in eliciting ALS-like pathology, namely non-transgenic C57BL6 mice (WT; n = 24 males; mean age 300 ± 20 days; Charles River Laboratories; Montreal, QC, Canada) and C57BL6 mice overexpressing human gene for TDP-43 (hTDP-43^WT^; n = 15 males and 12 females; mean age 254 ± 54 days; a kind gift from Christopher E Shaw, Kings College London).

The study consisted of three groups. The control group received intracerebroventricular (i.c.v.) infusion of pooled NALS-CSF (NALS group). Another group received i.c.v infusion of the pooled ALS-CSF and served as the diseased group (ALS group), whereas a third group received ALS-CSF infusion followed by oral administration of IMS-088, serving as the treatment group (ALS-IMS group).

### 2.2. Surgical Procedure and CSF Infusion

The i.c.v. delivery of CSF samples was done as previously reported [8]. Briefly, mice were anesthetized with 2% isoflurane in 100% oxygen at a flow rate of 2 L/min and placed in a stereotaxic apparatus. The sample administration with an Alzet osmotic mini-pump model 1002 (Durect, Cupertino, CA, USA) was carried out for 2 weeks at a rate of 0.25 μL/h. Then the behavioral experiments were conducted followed by euthanasia to investigate the molecular and histological pathology.

### 2.3. IMS-088 Administration

IMS-088 of approximately 98% purity was synthesized by Phyton Biotech (Delta, BC, Canada) from Withaferin-A derived from *Withania somnifera* biomass/root extract. IMS-088 was first dissolved in dimethylsulfoxide (DMSO) and diluted with 2% Tween 80 in saline at concentration of 5 mg/mL. The final concentration of DMSO was 5%. Transgenic hTDP-43^WT^ and non-transgenic (C57BL/6NCrl, Charles River) mice were anesthetized (Isoflurane) and administered with IMS-088 (30 mg/kg) by intragastric gavage, 48 h after the osmotic pump implantation for CSF delivery and then once daily for the following 13 days.

### 2.4. Behavioral Analyses

We performed an open field test (OFT) for assessment of the locomotor activity [8]. After being placed in the OFT chamber, the mice were video-recorded in trials of 5 min, using the VersaMax System and VersaPlot software (AccuScan Instruments, Inc., Columbus, OH, USA).

### 2.5. Tissue Collection

The mice were anesthetized with 10 μL/g pentobarbital (12 mg/mL) and euthanized by cervical dislocation. The spinal cord lumbar samples were dissected and stored at −80 °C for protein analysis. The tibialis anterior (TA) muscle tissues were collected and snap frozen to obtain 16 µm thick cryosections for Hematoxylin and Eosin (H and E) staining. For microscopy analysis, mice were transcardially perfused with PBS followed by fixation with 4% paraformaldehyde. Spinal cord, brain, and muscle samples were post-fixed overnight in 4% paraformaldehyde and equilibrated in a solution of PBS-sucrose (30%) for 48 h. Spinal cord lumbar region and brain tissues were then cut into 25 µm-thick sections with a Leica frozen microtome and stored at −20 °C. Muscle cryosections (16 µm thick) were prepared on a cryostat and stored at −20 °C.

### 2.6. Microscopy and Quantitative Analysis

H and E staining for muscle tissue was undertaken as previously reported [8]. Images were captured with the Leica DMI 6000B microscope (Leica Microsystems Inc., Richmond Hill, ON, Canada). The cross-sectional area of the muscles was measured with the particle analysis feature of ImageJ (FIJI). The area for each fiber was measured and a frequency distribution curve was produced. At least three sections per mouse were considered for analysis (n = 3 per group in triplicates).

Nissl staining was undertaken using fixed spinal cord sections according to a published protocol [8]. The quantification of neurons was carried out using a size-based filter above 250 μm^2^ to select motor neurons (n = 3 per group in triplicates).

For immunofluorescence microscopy, the brain and spinal cord sections were exposed to antigen retrieval 0.01 M citrate buffer (pH 6.0), washed twice with PBS, and blocked with 3% goat serum in 0.2 M TBS (pH 7.4). The sections were incubated with the primary antibodies (Table 1) overnight in TBS-Triton-X, followed with treatment with suitable fluorophore-tagged secondary antibody for 2 h (n = 3 per group in triplicates). The tissue sections were then washed with TBS-Triton-X and incubated with DAPI for 5 min, when applicable. The sections were then mounted with anti-fade mounting media and analyzed by confocal laser microscope (Nikon, Tokyo, Japan).

The integrated density of TDP-43 in the soma and nucleus of individual neurons was measured using the ImageJ (FIJI) as previously described [8]. The mitofilin staining was quantified in at least three sections per mouse using the particle analysis.

The muscles were stained for the neuromuscular junctions (NMJs) with α-Bungarotoxin antibody labeled with Alexa-594 (Invitrogen ThermoFisher, USA) and Anti Neurofilament (NF)-L antibody (MilliporeSigma, Oakville, ON, Canada) followed by a secondary antibody labeled with Alexa 488 (Table 1). The analysis was carried out by counting the number of fully and partially denervated NMJs from at least four sections per mouse and three mice per group. The average area of NMJs was calculated using the particle analysis of ImageJ (FIJI).

### 2.7. Immunoblotting

The snap-frozen spinal cord tissues were suspended in RIPA buffer. Insoluble and soluble fractions were obtained as previously described [8]. The protein extracts were fractionated by SDS-PAGE following by transfer on nitrocellulose membrane (LI-COR Biosciences, Lincoln, NE, USA). The membranes were probed with relevant antibodies (Table 1) followed by incubation with appropriate IR-conjugated (LI-COR Biosciences, USA), fluorescent-tagged, or HRP-conjugated secondary antibodies and visualized using Biorad Imager (Bio-Rad Laboratories, Hercules, CA, USA) (n = 3 per group in triplicates). The band intensities were measured with the Image lab 6.0 software (Bio-Rad Laboratories, USA) and normalized against appropriate loading control (GAPDH) or Revert total protein stain (LI-COR Biosciences, USA).

### 2.8. EDTA-Translational Affinity Purification (TRAP) Protocol

To determine the molecular signatures of neurons, we used the EDTA-TRAP approach with the hNfL-RFP mouse model previously described [8]. Briefly, on the 15th day post CSF infusion, freshly translated peptides were extracted from neuronal ribosomes from three mice per group and pooled to achieve optimal concentration for each group. From each pool, we used three technical replicates (n = 3 in technical triplicates). Briefly, lumbar spinal samples were collected and quickly homogenized in tissue lysis buffer. The samples were incubated with the anti-RFP agarose affinity resin (ChromoTek, Planegg-Martinsried, Germany) and the beads containing neuronal ribosomes were resuspended in EDTA-elution buffer to release the nascent peptides. The supernatant containing neuronal peptides was quantified with the Bradford assay and same amount of peptides was analyzed by Orbitrap fusion mass spectrometer (Thermo Fisher Scientific, San Jose, CA, USA).

### 2.9. Mass Spectrometry (MS) and Translatome Analysis

The peptide samples in triplicates (750 ng) from each group were prepared and processed for electrospray MS as described previously [8]. The analyses were carried out by the Proteomics Platform of the Eastern Quebec Genomic Center, CHU de Quebec, Canada. Quantification Spectra were searched against a mouse proteins database (UniprotKB—84,675 sequences). Only changes higher or lower than 1.5 folds with adjusted *p* < 0.05 were considered significantly dysregulated. The altered peptides were utilized to produce PCA plots, comparative heatmaps, and Venn diagrams across the groups. Peptides (genes) differentially expressed in the ALS v/s ALS-IMS group were further used to generate biological networks using the Gene Ontology (GO) cellular component in the FunRich analysis software (version 3.13) [16]. Only significantly enriched leading terms with corrected *p*-value (*p* < 0.0; Bonferroni method) were considered for the comparison. Further, to understand the processes altered by the administration of IMS-088 to ALS-CSF-infused animals, we generated an enrichment network using the Reactome database with the help of the online tool called Network Analyst [17].

### 2.10. Study Approval

All experiments were approved by the animal care ethics committee of Laval University (2016060-2) in accordance with the Canadian Council on Animal Care. CSF samples were collected at ALS Clinic of CHU de Quebec after written consent in accordance with the guidelines of the institutional human ethics committee.

### 2.11. Statistics

Statistical analyses were carried out with GraphPad Prism 8.0 (GraphPad, Inc., San Diego, CA, USA), using a one- or two-way ANOVA with Tukey’s post-hoc test or Bonferroni post-hoc test. The results are presented as mean ± SEM and were considered significant when * *p* < 0.05, ** *p* < 0.01, *** *p* < 0.001, **** *p* < 0.0001 NALS v/s ALS, NALS v/s ALS-IMS, ALS v/s ALS-IMS; or $ *p* < 0.05, $$ *p* < 0.01, $$$ *p* < 0.001, $$$$ *p* < 0.0001 ALS v/s ALS-IMS, as specified.

## 3. Results

### 3.1. IMS-088 Treatment Ameliorates Motor Function of Mice Infused via i.c.v. with ALS-CSF

**Figure 1:**
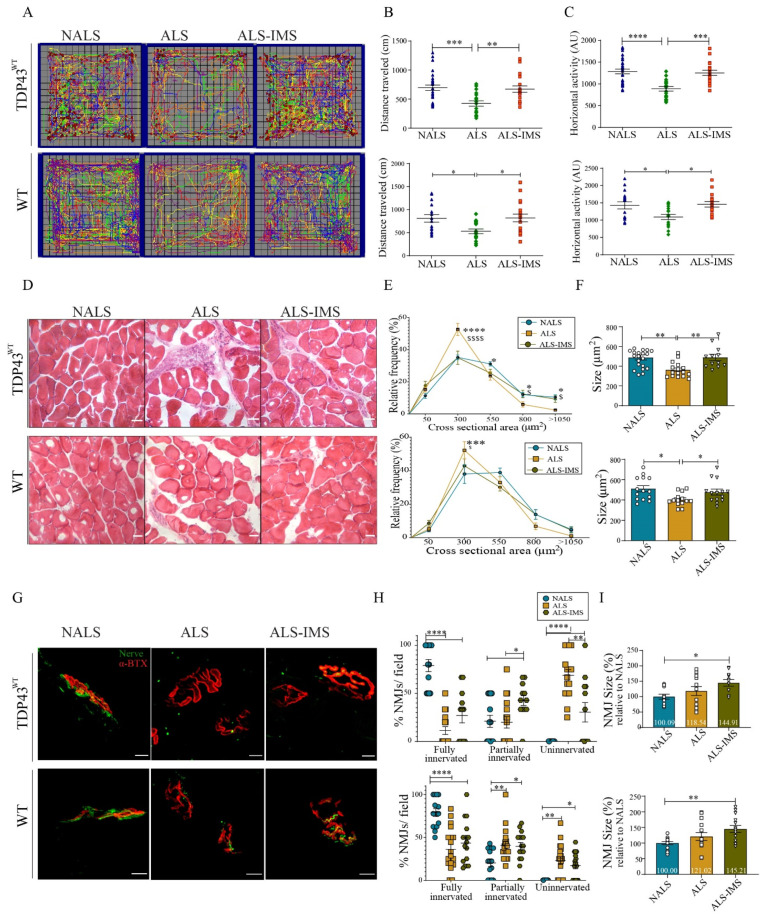
IMS-088-mitigated motor dysfunction and muscular pathology induced by i.c.v. infusion of ALS-CSF. (**A**) Panel representing open field test (OFT) analysis for transgenic (hTDP-43^WT^; n = 6) and non-transgenic (WT; n = 8) mice. Each mouse trajectory is represented by a distinct color. The locomotor pattern was quantified for total distance traveled (**B**) and horizontal activity (**C**) by the mice during OFT. The blue triangles, green diamonds and red squares represent mice infused via i.c.v. with NALS-CSF, ALS-CSF and ALS-CSF plus oral IMS-088, respectively. (**D**) Panel representing structural changes in the H&E-stained muscle tissues, with graphs depicting the effect on the cross-sectional area (**E**) and average size (**F**) of the muscle fibers (n = 3; scale bar = 50 µm). Panel (**G**) represents the changes in the NMJs of the hTDP-43^WT^ and WT mice following different treatments. Graph panel (**H**) highlights changes in innervation of NMJs, while graph panel (**I**) denotes the changes in the average size of NMJs (n = 3; scale bar = 10 µm). The circles, squares and triangles in graphs represent mice infused via i.c.v. with NALS-CSF, ALS-CSF and ALS-CSF plus oral IMS-088, respectively. Data are mean ± SEM. (* *p* ≤ 0.05, ** *p* ≤ 0.01, *** *p* ≤ 0.001, **** *p* ≤ 0.001 NALS v/s ALS; and $ *p* ≤ 0.05 and $$$$ *p* ≤ 0.001 ALS v/s ALS-IMS) and fold changes are calculated compared to NALS.

The hTDP-43^WT^ transgenic mice at 8 months of age were infused via i.c.v. with CSF samples from ALS patients or non-ALS controls (NALS) using mini-osmotic pumps. At 48 h after osmotic pump implantation, a group of mice (n = 6) infused with ALS-CSF received IMS-088 (30 mg/kg) by intragastric gavage and then once daily for another 13 days. The other group (n = 8) infused with ALS-CSF received vehicle treatment. At day 15 after pump implantation, we monitored the locomotory behavior of hTDP-43^WT^ transgenic mice and C57BL6 WT (B6^WT^) mice under different experimental conditions through OFT analysis (Figure 1A–C). IMS-088 administration was found to rescue the loss of locomotor ability in the mice infused with ALS-CSF, as measured through total distance traveled (Figure 1B) as well as horizontal activity (Figure 1C). The OFT analysis also revealed the enhanced vulnerability of hTDP-43^WT^ mice to ALS-CSF treatment when compared to the WT mice. ALS-CSF infusion reduced the locomotor ability in the hTDP-43^WT^ mice by ~40%, whereas the reduction in the locomotor ability of B6^WT^ mice in response to ALS-CSF was ~23% (Figure 1B). IMS-088 treatment fully restored the locomotor ability in the hTDP-43^WT^ and B6^WT^ mice. Similarly, the reduction in horizontal activity in response to ALS-CSF for the hTDP-43^WT^ and WT mice was 31% and 17%, respectively, with a rescue percentage with IMS-088 for both mice being 97% (Figure 1C).

The changes observed in the locomotor patterns was also reflected in the muscle pathology across the groups. H and E staining revealed reduction in the cross-sectional area (Figure 1D,E), as well as the average size of the muscle fibers observed in response to ALS-CSF, which was significantly ameliorated by IMS-088 administration (Figure 1F). Further, the neuromuscular junctions (NMJs) were examined following immunostaining of the presynaptic (anti-NFL, green) and postsynaptic (anti Btx, red) components (Figure 1G). We performed an analysis of the fully or partially innervated, as well as the denervated NMJs in each experimental group. While ALS-CSF infusion in hTDP-43^WT^ mice caused the complete denervation of 70% NMJs (ALS-CSF vs. NALS-CSF), IMS-088 mitigated the effects of ALS-CSF on NMJs by reducing full denervation to only 25% (ALS-IMS vs. NALS). We also observed significant increase of partial innervation by IMS-088 treatment (~23% ALS-IMS vs. ALS, *p* = 0.0256), suggesting that the compound was able to prevent NMJs from total loss of function (Figure 1H).

However, in the B6^WT^ mice we observed a relatively milder muscular pathology triggered by ALS-CSF infusion with a partial and complete loss of innervation amounting to ~44% (ALS vs. NALS), which was only partially reversed by IMS-088 administration (Figure 1H). It is noteworthy that there was a significant increase in the NMJ size for both mouse models in response to IMS-088 (Figure 1I), suggesting a compensatory effect of IMS-088 treatment on the synaptic plasticity within the surviving NMJs [18]. These data combined with the OFT observations of motor impairment further validated the strong phenotype of the hTDP-43^WT^ mice model with ALS-CSF infusion. Therefore, we have focused on the hTDP-43^WT^ mouse model for subsequent experiments, except for analyses of the neuronal translational profiles (Figure 5) which included WT mice to understand the specific effects of CSF on the less susceptible neuronal tissues.

### 3.2. IMS-088 Mitigated Spinal Neuronal Loss and Neurofilament Disorganization in hTDP-43^WT^ Mice Infused with ALS-CSF

**Figure 2:**
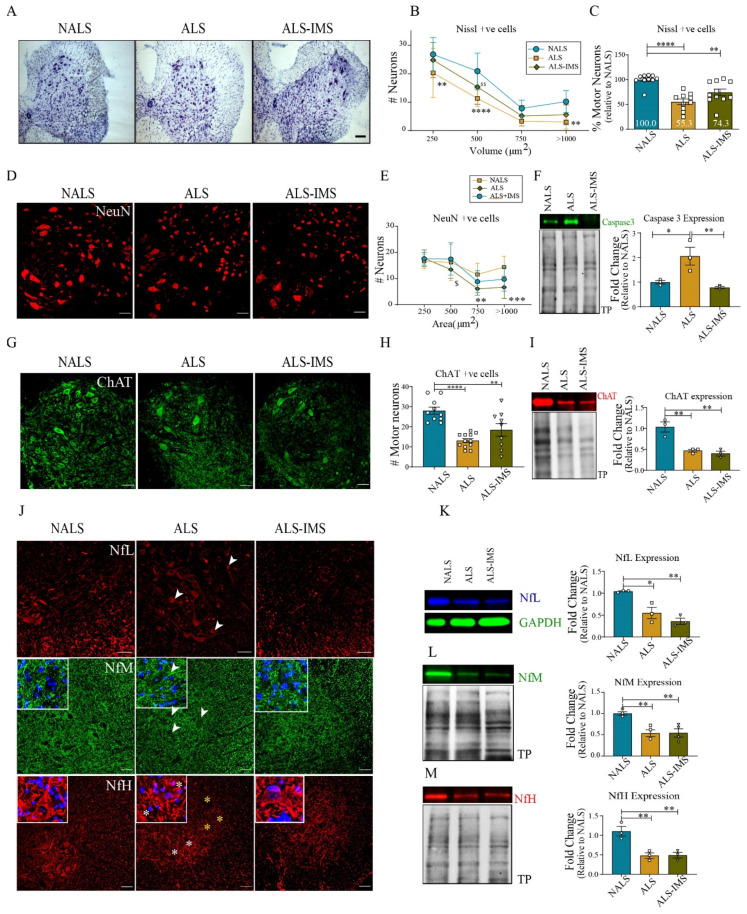
IMS-088 administration reduced loss of spinal neurons and neurofilament disorganization in mice exposed to ALS-CSF. Spinal cord sections stained with Cresyl violet (**A**) and quantified (**B**,**C**) as well with NeuN (**D**,**E**), respectively. Scale bar = 50 and 20 µm, respectively. Expression patterns and quantification of the immunoblots for Caspase-3 protein (**F**) in the spinal cord lysates. (**G**,**H**) represent the spinal cord sections stained and quantified for ChAT expression and (**I**) represents the changes in ChAT expression levels across the spinal cord lysates of the various experimental groups. (**J**) represents the spinal cord sections stained for the neurofilament proteins NfL (red), NfM (green), and NfH (Red). Note the changes in the abnormal localization of neurofilament proteins in the ALS group, particularly the aggregated patterns of NfM and NfL (white arrowheads, inset image) in neuronal cell bodies, and reduced (yellow asterisk) or unevenly distributed NfH (white asterisks) immunostaining in the neuronal soma. (**K**–**M**) represent the expression patterns and quantification for NfL, NfM, and NfH proteins, respectively, in the immunoblots from mice spinal cord lysates (n = 3 in triplicates; scale bar = 20 µm; fold changes are calculated compared to NALS). The circles, squares and triangles in graphs represent mice infused via i.c.v. with NALS-CSF, ALS-CSF and ALS-CSF plus oral IMS-088, respectively. Data are mean ± SEM. (* *p* ≤ 0.05, ** *p* ≤ 0.01, *** *p* ≤ 0.001, **** *p* ≤ 0.001, $ *p* < 0.05, $$ *p* < 0.01).

At day 15 after initiation of i.c.v. infusion of CSF samples in hTDP-43^WT^ mice, we counted motor neurons based on the size (>250 µm^2^) in the spinal cord sections stained with Cresyl Violet (Figure 2A–C) or with NeuN (Figure 2D,E). The results revealed a protective effect of IMS-088 treatment. The number of neurons was 19% higher (*p* = 0.0580) in spinal cord samples from IMS-088-treated mice when compared to vehicle-treated mice, although the result marginally missed the statistical significance threshold (Figure 2C). In accordance, IMS-088 treatment prevented the induction of Caspase-3 expression by ALS-CSF as revealed by immunoblotting (Figure 2F). The motor neuron counts by immunofluorescence microscopy with anti-ChAT antibody (Figure 2G,H) revealed a trend of increase in ChAT-positive neurons in the IMS-088-treated group when compared to the vehicle-treated group (~29% higher in ALS-IMS-088 v/s ALS-vehicle). However, immunoblot analysis showed that IMS-088 treatment did not prevent the downregulation of ChAT levels triggered by exposure to ALS-CSF (Figure 2I).

To further understand the extent of neuronal damage in response to ALS-CSF, as well as the effectiveness of IMS-088 in ameliorating neuronal pathology, we examined the distribution by immunofluorescence microscopy and expression levels of neurofilament NfL, NfM, and NfH proteins by immunoblotting of spinal cord extracts. ALS-CSF infusion in hTDP-43^WT^ mice provoked a mislocalization of NfL and NfM in the soma of neurons (Figure 2J, white arrowheads), with depletion of these proteins in neurites and axons. Remarkably, IMS-088 treatment rescued in part the misdistribution of Nfl and NfM proteins triggered by ALS-CSF exposure. For NfH immunodetection, we used an antibody that detects both phosphorylated and dephosphorylated NfH-chain (Millipore #MAB5266). Immunostaining of the spinal cord with this antibody yielded a NfH immunostaining in neuronal soma and axons in the control NALS-CSF-infused mice (Figure 2J). In contrast, the ALS-CSF-treated mice exhibited reduced NfH immunostaining in the soma of neurons (Figure 2J). The perikaryal NfH distribution was recovered by IMS-088 administration (Figure 2J). When analyzed by immunoblotting, the spinal cord levels of NfL (Figure 2K), NfM (Figure 2L), and NfH (Figure 2M) were decreased by ~50% in response to ALS-CSF infusion in TDP-43^WT^ mice. However, treatment with IMS-088 failed to restore normal levels of NF proteins. Our interpretation is that IMS-088 was able to alleviate the abnormal distribution of NF proteins in soma and axons triggered by ALS-CSF exposure, but there was some irreversible loss of NF proteins due to neuronal death after ALS-CSF infusion, especially before initiation of treatment with IMS-088 at two days after beginning of i.c.v. infusion of ALS-CSF (Figure 2A–H).

### 3.3. IMS-088 Treatment Rescued TDP-43 Proteinopathy Associated with ALS-CSF Infusion

**Figure 3:**
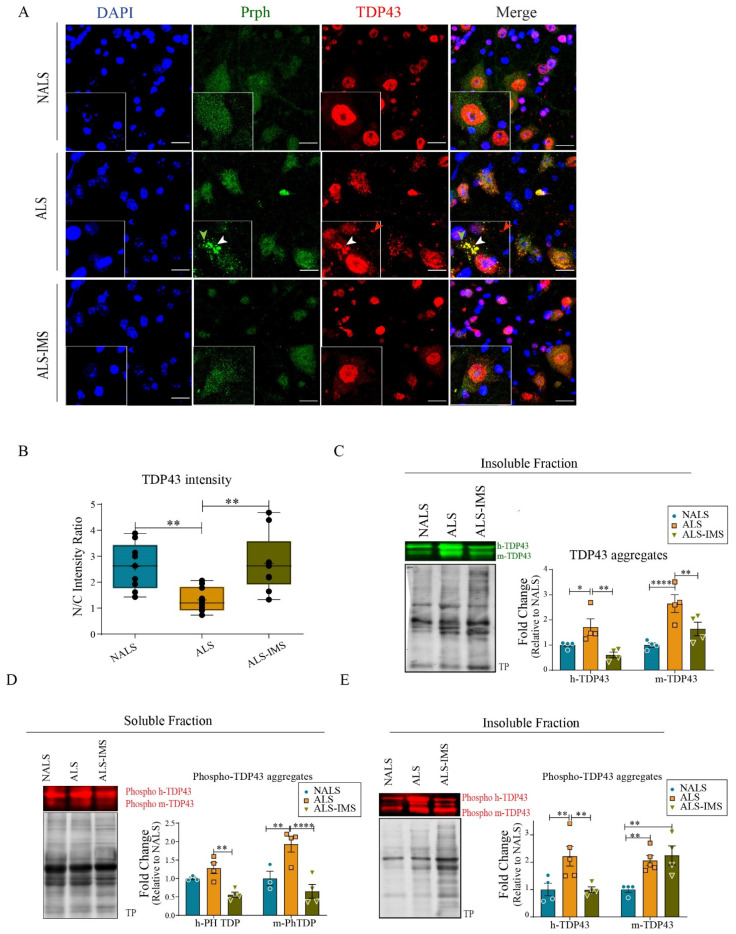
IMS-088 treatment prevented TDP-43 proteinopathy triggered by ALS-CSF exposure. (**A**) Representative image panel showing cellular localization of TDP-43 (red) and peripherin (Prph, green) in the hTDP-43^WT^mice in different treatment groups. Note the presence of cytoplasmic TDP-43 aggregates with (white arrowhead) or without Prph (red arrowhead) in the magnified ALS inset image. Also note the presence of peripherin aggregates independent of TDP-43 aggregates (green arrowhead). IMS-088 treatment ((**A**), lower panel) decreased the formation of cytoplasmic TDP-43 and peripherin aggregates. Graph (**B**) depicts the reversal of mislocalization of TDP-43 in response to IMS treatment of the ALS-CSF administered mice. (**C**–**E**) represent qualitative and quantitative observations for the immunoblots for aggregated TDP-43 in the insoluble fraction (**C**) as well as phosphorylated TDP-43 present in the soluble (**D**) and insoluble (**E**) fractions of mice spinal cord lysates. (n = 3, in triplicates; scale bar = 20 µm; fold changes are calculated compared to NALS). * *p* < 0.05, ** *p* < 0.01, **** *p* < 0.0001.

As abnormal cytoplasmic aggregation of TDP-43 is a most prominent pathological feature of ALS [19,20], we examined the effect of IMS-088 on TDP-43 proteinopathy triggered by ALS-CSF infusion in hTDP4^WT^ mice. In the ALS-CSF group treated with vehicle, immunofluorescence microscopy with anti-TDP-43 antibody revealed cytoplasmic mislocalization and aggregation of TDP-43 in spinal neurons (Figure 3A, red). Remarkably, oral administration of IMS-088 rescued the TDP-43 pathology triggered by ALS-CSF infusion in hTDP-43^WT^ mice. Quantification of the nuclear to cytoplasmic ratio of TDP-43 immunofluorescence signal further corroborated the rescue of protein mislocalization by IMS-088 treatment. In the IMS-088-treated group, the nuclear to cytoplasmic ratio of TDP-43 was significantly higher than the vehicle-treated group, and comparable to that of the NALS group (Figure 3B). Furthermore, RIPA-insoluble fractions from the spinal lysates were immunoblotted with anti-TDP-43 antibody to assess the level of aggregated TDP-43. Two distinct TDP-43 bands corresponding to the endogenous mouse TDP-43 aggregates (~43 kDa) and to the Myc-tagged human TDP-43 (~45 kDa) were detected in the insoluble fractions (Figure 3C). The levels of both TDP-43 species in the insoluble fractions were increased in response to ALS-CSF, whereas IMS-088 treatment reduced levels of insoluble TDP-43 to those found in NALS group (Figure 3C).

Phosphorylation of TDP-43 is another feature of ALS pathology [21]. Using antibody against phospho-TDP-43 (Cosmobio P01), immunoblotting of spinal cord extracts revealed that ALS-CSF infusion in hTDP-43^WT^ mice increased the levels of phosphorylated TDP-43 species (human and mouse) recovered in the soluble and insoluble fractions (Figure 3D,E). IMS-088 treatment of ALS-CSF-infused hTDP-43^WT^ mice restored levels of phospho-hTDP-43 to those found in NALS-CSF-infused hTDP-43^WT^ mice.

Increased levels of peripherin expression and aggregation in response to ALS-CSF have been reported previously [8,22]. Here, in ALS-CSF-infused hTDP-43^WT^ mice, peripherin accumulations were also detected by immunofluorescence in spinal motor neurons (Figure 3A, green arrowhead). Interestingly, subsets of peripherin inclusions co-localized with TDP-43 immunostaining (red) in the merge picture suggesting interactions between some pathological forms of peripherin and TDP-43 in the neuronal cytoplasm (Figure 3A, yellow aggregates in the merge picture).

### 3.4. Reduced Neuroinflammation in Mice Treated with Withaferin-A Derivative

**Figure 4:**
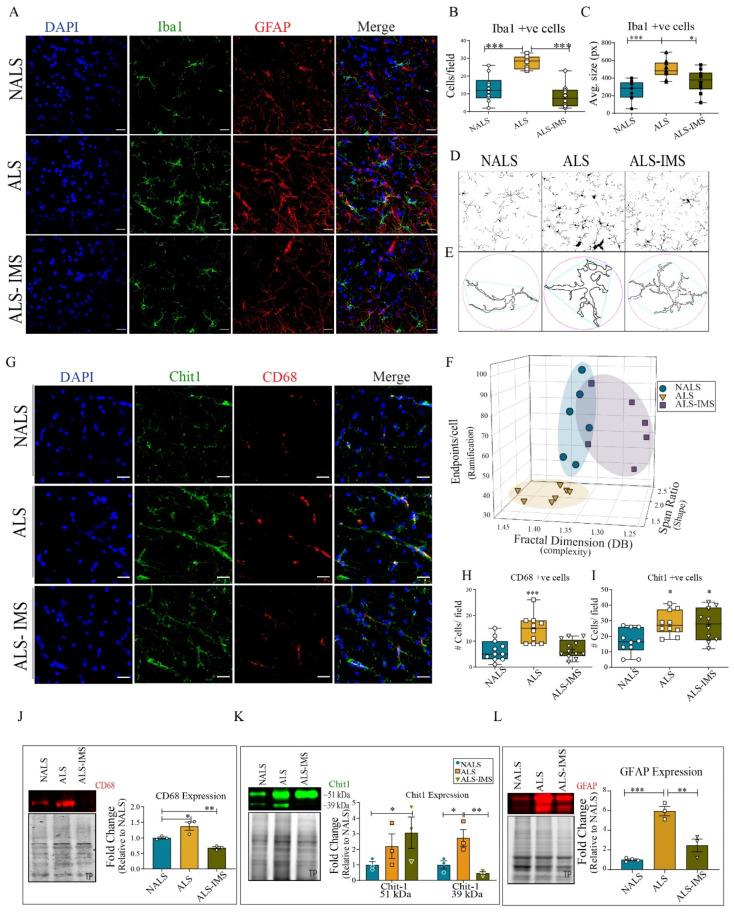
IMS-088 mitigated the neuroinflammatory responses to ALS-CSF. (**A**) Representative confocal images of the spinal cord sections of hTDP-43^WT^ mice stained with DAPI (blue), Iba1 (green), and GFAP (red). The number (**B**) and average size (**C**) of Iba1-positive cells per fields was quantified to reveal the effects of different treatments. Further, the dynamic, structural changes in microglial morphology were analyzed (**D**,**E**) to assess the shape complexity and ramification status of microglial cells under different experimental conditions (**F**). (**G**) Representative image panel of the spinal cord sections stained with DAPI (blue), Chit1 (green), and CD68 (red) across various experimental conditions. The numbers of Chit1-positive (**H**) and CD68-positive (**I**) cells were counted across each group to further assess the activation differences. Expression patterns and quantification of the immunoblots for CD68 (**J**), Chit1 (**K**), and GFAP (**L**) proteins in the spinal cord lysates were further analyzed. Fold changes are calculated when compared to NALS (n = 3 in triplicates; scale bar = 20 µm). The circles, squares and triangles in graphs represent mice infused via i.c.v. with NALS-CSF, ALS-CSF and ALS-CSF plus oral IMS-088, respectively. Data are mean ± SEM. (* *p* ≤ 0.05, ** *p* ≤ 0.01, *** *p* ≤ 0.001).

Since *Withania somnifera* extracts and Withaferin-A exhibited anti-inflammatory effects in various models of ALS and FTLD [10,11,23], we investigated the effect of the derivative IMS-088 on neuroinflammation in ALS-CSF-infused hTDP-43^WT^ mice (Figure 4). Spinal cord sections were immunostained for the microglial marker Iba1 (Figure 4A, green) and astroglial marker GFAP (Figure 4A, red). There was an increase in the number of Iba1- and GFAP-positive cell bodies in the ALS-CSF-infused group, which was rescued by IMS-088 treatment. Quantification of the Iba1 immunostaining further validated the increased number (Figure 4B) and average size (Figure 4C) of microglial cells in the ALS-CSF-infused group. IMS-088 administration reverted the microgliosis triggered by ALS-CSF exposure (Figure 4C).

Moreover, we performed a thorough analysis of morphological changes of the microglia population to further understand the nature of activation. A detailed study by Morrison et al. [24] attempted to associate morphological changes in microglia with their activation status as well as spatial and temporal relevance in their model of traumatic brain injury [24]. Taking this study as a reference, we compared the morphological status of microglia in our experiment (Figure 4D–F). We used skeleton (Figure 4D) and fractal analyses (Figure 4E), to determine the endpoints, fractal dimensions, and span ratio for each cell, which denoted the state of its ramification, complexity, and shape, respectively. Please note that although the skeleton analysis for endpoints was performed without applying the ‘fill holes’ feature while skeletonizing the images, the image has made use of the feature to highlight the morphological difference across the groups (Figure 4D). The results indicated patterns of ALS replicates distinct from NALS-CSF and ALS-CSF-IMS-088 groups (Figure 4F); with cells acquiring more de-ramified hypercomplex or de-ramified rod-shaped morphology in response to ALS-CSF. The microglial activation of the ALS-CSF-treated group loosely resembled that of the microglial diversity on day 7 at the site impact of a brain injury representing a state of chronic inflammation [24]. Although there is some overlap between NALS-CSF and ALS-CSF-IMS-088 replicates, the ALS-CSF-IMS-088 group had distinctive microglial morphology reminiscent of a mix of ramified and rod-shaped morphology in contrast to the ramified and less complex morphology seen in the NALS-CSF group (Figure 4F).

To investigate the activation status of the microglial cells, we further analyzed the spinal cord for the activation marker CD68 and ALS-specific microglial protein Chit1 [25] (Figure 4E). Quantification of the results revealed an increase of CD68-positive cells in spinal cord sections of ALS-CSF-treated group (Figure 4G,H) which was corroborated with an increase of CD68 levels by immunoblotting of spinal cord lysates (Figure 4J). The CD68 increase in ALS-CSF-infused mice was rescued by IMS-088 administration. We also observed by immunofluorescence an increase in the Chit1-positive cells in response to ALS-CSF infusion, but this remained unchanged by IMS-088 administration (Figure 4G,I). The immunoblot analysis for Chit1 revealed increased expression of the precursor form of Chit1 (~51 kDa) in both vehicle- and IMS-088-treated ALS-CSF groups (Figure 4K). However, the increased expression of its enzymatically active form (~39 kDa) [26] in response to ALS-CSF was mitigated by IMS-088 administration (Figure 4K).

Concomitant to microglial activation, we also observed astroglial activation in the form of increased GFAP-positive cells (Figure 4A) as well as expression levels of GFAP (Figure 4L), both of which were rescued by IMS-088 administration. These results convincingly demonstrate the anti-inflammatory effects of IMS-088 administration in this mouse model based on ALS-CSF infusion.

### 3.5. Proteome of Spinal Neurons Reveals Multiple Pathways Associated with Therapeutic Effects of IMS-088 in ALS Pathology

To further investigate in this mouse model the effects of IMS-088 on the molecular profiles/proteome of neuronal cells, we used an EDTA-TRAP protocol involving the immunoprecipitation of polyribosome complex with the attached mRNA and nascent peptide chains in a cell-specific manner. We have previously reported the altered translational profiles of spinal neurons from doubly transgenic hTDP-43^WT^ whereby hNf-RFP mice after i.c.v. infusion of ALS-CSF express HA-RFP1-tagged rpl10 ribosomal protein [8]. Here, we have examined the translational profiles of single transgenic hNfL-RFP mice infused with NALS-CSF, ALS-CSF, or ALS-CSF with IMS-088. For each group, we pooled spinal cords (lumbar region) from three different mice to achieve optimal protein levels for the experiment (n = 3). The newly translated peptides bound to ribosomes were extracted and analyzed by mass spectrometry. For the analysis, three technical replicates were taken from each pool.

**Figure 5:**
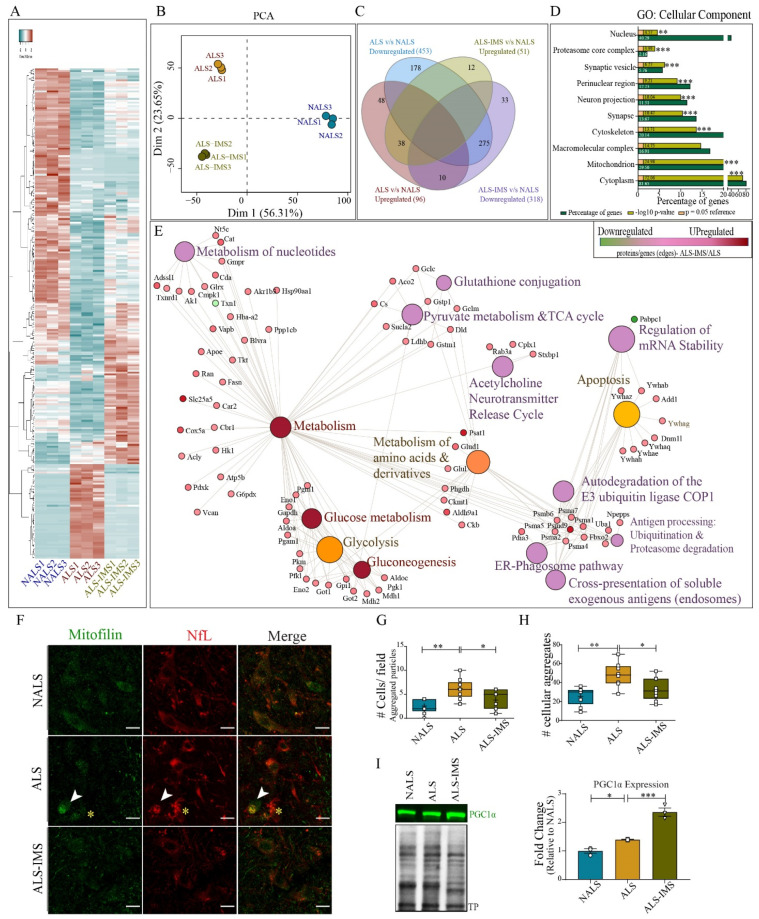
IMS-088 treatment partially rescued the neuronal translational defects induced by infusion of ALS-CSF. (**A**) Heatmap and (**B**) principal component analysis showing the pattern of regulation of the neuronal translatome in the groups. (**C**) Venn diagram of the proteins differentially regulated in ALS and ALS-IMS group when compared to NALS (disease control) group. (**D**) Graph depicting cellular component of the processes (GO processes) specifically altered by IMS-088 treatment of the ALS-CSF-administered mice (ALS v/s ALS-IMS). (**E**) Network depicting significantly altered cellular processes or nodes (Reactome) with corresponding upregulated (pink) or downregulated (green) proteins or edges (ALS v/s ALS-IMS). Panel (**F**) shows spinal cord sections stained with mitofilin (green) and NfL (red). (**G**) represents the analysis of total number of cells per field with aggregated pattern of mitofilin, while (**H**) denotes the analysis of overall aggregates per field counted using particle analysis feature of ImageJ (FIJI). (**I**) shows expression patterns and quantification of the immunoblot for PGC1α from mouse spinal cord lysates (n = 3, in triplicates). * *p* < 0.05, ** *p* < 0.01, *** *p* < 0.001.

A heat map produced from the altered proteins revealed significant variations in the translational patterns of ALS-CSF groups compared to NALS-CSF, where most of the significantly altered proteins were downregulated (Figure 5A) in ALS replicates. Many of the proteins altered in ALS groups reverted to levels comparable to the NALS group upon IMS-088 administration (Figure 5A). The difference between the translational profiles was further evident through principal component analysis (PCA), where we observed distinctive patterns of protein translation in each group (Figure 5B). Both, the heatmap and the PCA analysis suggested an intermediate translational profile of ALS-IMS, reminiscent of the partial and complete rescue that we observed with the neurodegeneration, IF disorganization, and TDP-43 pathology (Figure 2 and Figure 3). Out of 453 proteins downregulated in ALS groups (ALS v/s NALS), 178 proteins were unique to ALS while 275 others were upregulated in the IMS-088 group (ALS-IMS v/s NALS), suggesting their complete rescue (Figure 5C). Similarly, only 38 out of 96 proteins upregulated in ALS (ALS v/s NALS) remained upregulated in the IMS-088 group as well (ALS-IMS v/s NALS), suggesting a complete rescue in the translation of nearly two-thirds of the upregulated proteins (Figure 5C). Interestingly, there were ~50 proteins uniquely altered (up or downregulated) in the IMS group (ALS-IMS v/s NALS), possibly hinting towards initiation of unique reparative processes (Figure 5C). To further expand on the understanding of the functions affected by these dynamic translational changes, we resorted to the categorization of the proteins specifically altered in the IMS groups in comparison to the ALS groups (ALS-IMS v/s ALS) into their respective cellular component and biological functions (Figure 5D,E). The GO cellular component terms corresponding to the dysregulated proteins indicated the involvement of cytoskeletal, mitochondrial, nuclear, synaptic, and proteasomal proteins in the rescue process mediated by IMS (ALS-IMS v/s ALS) (Figure 5D). Further, analysis of the biological processes (Reactome) revealed multiple pathways significantly implicated in the IMS-088-mediated mitigation of the ALS-CSF pathology converging on to glucose, nucleotide, and amino acid metabolism (glycolysis, gluconeogenesis, pyruvate metabolism, and Tricarboxylic acid cycle) (ALS-IMS v/s ALS). We also observed alteration of a class of proteins involved in multiple processes and pathways at the convergence of the ubiquitination–proteasome degradation, endosomal, and ER-phagosome pathway, indicating functional relevance of IMS-088 effects in pathways related to cellular and extracellular waste removal, toxin clearance, and antigen representation (ALS-IMS v/s ALS) (Figure 5E) [27]. Other significantly rescued processes involved apoptosis, reference acetylcholine neurotransmitter release cycle, regulation of mRNA stability, and glutathione conjugation (ALS-IMS v/s ALS) (Figure 5E).

These observations predominantly hinted towards the potential of IMS-088 to restore the imbalance in the energy homeostasis occurring in the hNfL-RFP mice exposed to ALS-CSF. Immunostaining of spinal cord sections of hTDP-43^WT^ mice infused with NALS-CSF or ALS-CSF with or without IMS-088 further validated these observations (Figure 5F). While we observed an evenly localized punctate pattern of mitofilin staining (green) across all neurons in the NALS-CSF group, the staining pattern was uneven across the ALS-CSF group, with some neurons staining more intensely than others within the same field. In the stressed neuronal subpopulation with aggregated NfL in the soma (Figure 5F, red, white arrowheads, and yellow asterisks), the mitofilin staining was either heavily aggregated (Figure 5F, white arrowheads) or weekly stained (Figure 5F, yellow asterisks). IMS-088 administration appeared to reverse both aggregated mitofilin as well as aggregated NfL in the spinal neurons of mice infused with ALS-CSF. Moreover, analysis of the mitofilin aggregates using the particle analysis feature of ImageJ (FIJI) revealed an increase in the total number of cells/fields containing aggregates (Figure 5G), and the number of aggregates in these cells (Figure 5H) was significantly higher in the ALS group when compared to either NALS or ALS-IMS groups. The dysregulated mitochondrial physiology observed in the ALS group was also accompanied by an increase in the expression of PGC1α (~1.5 fold, ALS v/s NALS; Figure 5I), a transcription coactivator central to mitochondria homeostasis [28]. Interestingly, there was also a significant upregulation in the PGC1α expression (~2.4 fold, ALS-IMS v/s NALS; Figure 5I) which exceeded the upregulation observed in the ALS group (~1.7 fold, ALS-IMS v/s ALS; Figure 5I). Since PGC1α is upstream to various processes regulating mitochondrial physiology, these findings hinted towards a significant role of the rearrangement of the mitochondrial physiology in the protection conferred by IMS-088 in our present study.

### 3.6. Ubiquitin–Proteasome Pathway Was Rescued by IMS-088 Administration

**Figure 6:**
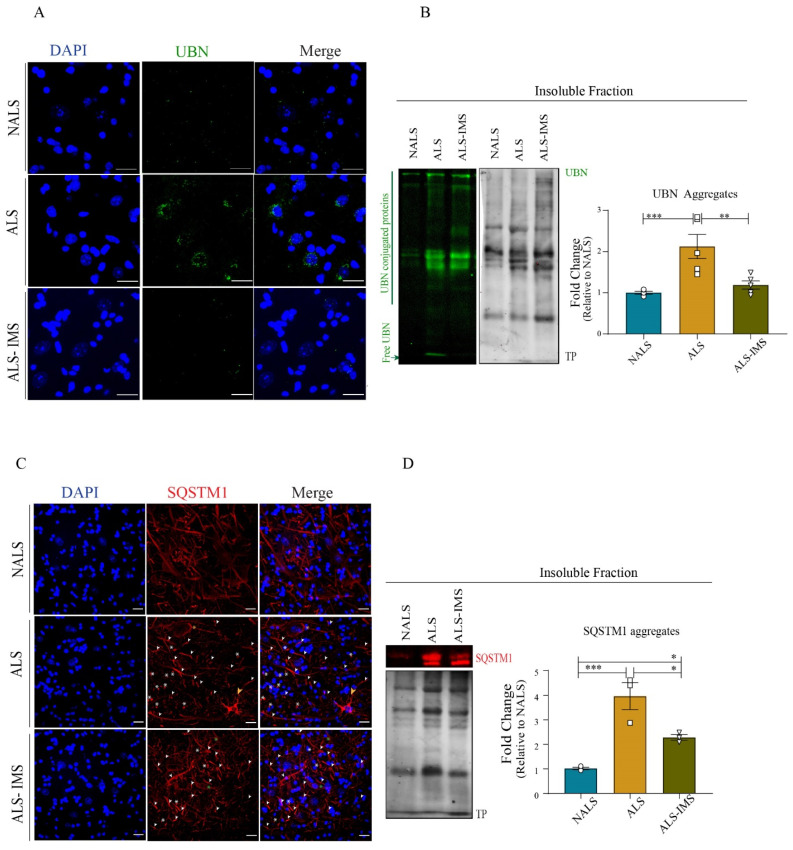
IMS-088-mitigated ALS-CSF-induced pathology by regulating ubiquitin–proteasome pathways. (**A**) Representative image panel showing spinal cords of the hTDP-43^WT^ mice immunostained for ubiquitin (UBN). (**B**) represents qualitative and quantitative observations for the immunoblots for aggregated UBN in the insoluble fraction of the mice spinal cord lysates. (**C**) depicts the localization of Sequestosome1 (SQSTM1) in different treatment groups. Note the increased SQSTM1 inclusions in different cell types in the ALS group (white arrowheads) in spinal cord sections of mice across different groups and (**D**) represents qualitative and quantitative analysis of the immunoblots for SQSTM1 aggregates in the insoluble fraction of the spinal cord lysates (n = 3, in triplicates; Sscale bar = 20 µm). * *p* < 0.05, ** *p* < 0.01, *** *p* < 0.001.

Our observations of altered ubiquitin-mediated degradation pathways converging with phagosomal and endosomal pathways in the neuronal translatome led us to investigate the levels of ubiquitin (UBN) as well as sequestostosome1 (SQSTM1), a marker of proteasomal degradation [29,30]. Immunostaining for UBN revealed an aggregated pattern of the protein in the spinal cord sections in response to ALS-CSF. These UBN aggregates were attenuated upon treatment with IMS-088 (Figure 6A). To confirm this, we investigated the levels of UBN protein in the RIPA-insoluble fractions obtained from the spinal lysates. We observed increased UBN moieties as well as expression in the lane corresponding to ALS-CSF fractions, which included free UBN as well as UBN-conjugated proteins sequestered for degradation. We also observed more ubiquitinated bands in the lane corresponding to ALS fractions. Both the UBN conjugated moieties as well as the intensity of free UBN expression were attenuated in the IMS group compared to the ALS group (Figure 6B).

Similar to the UBN pathology, we observed peculiarities in SQSTM1 immunostaining in ALS-CSF groups (Figure 6C). The neuronal soma in response to ALS-CSF reflected unevenly distributed SQSTM1 staining (Figure 6C, yellow asterisk), SQSTM1 puncta (Figure 6C, white asterisk), and aggregate-laden neuronal processes (Figure 6C, white arrowheads). Interestingly, we observed sporadically non-neuronal cell bodies that were heavily laden with SQSTM1 aggregates (Figure 6C, yellow arrowheads) exclusively in the ALS-CSF group. In comparison, IMS-088 treatment conferred a partial recovery as the SQSTM1 pathology was mainly limited to axonal aggregation, while the non-neuronal-containing aggregates were absent, the large puncta became sparse, and the staining across neuronal soma was more evenly spread (Figure 6C, green asterisks). The partial restoration of SQSTM1 expression in the IMS-group as compared to the ALS group was significant, as confirmed by the quantitative analysis of the immunoblots from the RIPA-insoluble fractions across the groups (Figure 6D). These results indicated that both UBN–proteasome and the autophagy pathways were perturbed by ALS-infusion and that the restoration of these pathways was one of the modes of action of IMS-088-mediated protection.

## 4. Discussion

In a previous study, we reported that the CSF from sporadic ALS cases can transmit via i.c.v. infusion in hTDP-43^WT^ mice an ALS-like disease with TDP-43 proteinopathy, neuroinflammation, cytoskeletal dysfunction, and metabolic dysfunction [8]. The toxic factors in the CSF of sporadic ALS patients responsible for the disease transmission remain to be elucidated. A report by Wong et al. identified apolipoprotein B-100 as a toxic agent in the ALS-CSF of disease transmission in mice by intrathecal injection [31]. However, other potential toxic factors cannot be excluded. Here, our results revealed that transgenic mice expressing human TDP-43^WT^ are more vulnerable to toxicity of ALS-CSF than normal mice, especially in regard to extent of muscle denervation (Figure 1H). This would support a concept of TDP-43 oligomer seeding and propagation by TDP-43 species from ALS-CSF in a prion-like mechanism [5]. In any case, our finding of protective effects of an analog of Withaferin-A (IMS-088) in pathogenesis transmitted by ALS-CSF infusion suggests a key role of NF-κB signaling in degenerative pathways.

Oral administration of IMS-088 ameliorated the motor function of mice infused with ALS-CSF, as evident from analyses of locomotor activity and muscle integrity. IMS-088 treatment led to increased partial innervation in the ALS-CSF group as compared to the vehicle-treated ALS-CSF group. Concomitantly, we also observed hypertrophy of NMJs in response to IMS-088 (Figure 1I), a phenomenon also associated with physical exercise and recovery from peripheral nerve injuries, and which can be modulated by changes in the skeletal muscle physiology [32,33]. A partial restoration of integrity and enhanced plasticity of NMJs by IMS-088 likely contributed to the rescued motor behavior of the mice infused with ALS-CSF. A direct effect of the drug in muscle is possible as Withaferin-A was found to promote mitochondrial function in skeletal muscle [34].

In the present study, we started IMS-088 treatment two days after the initiation of i.c.v. ALS-infusion in mice, allowing the pathology to propagate for 48 h before treatment. This could explain a partial rescue of treatment on disease phenotypes. Accordingly, IMS-088 cannot restore irreversible damage due to neuron death and loss of NMJs. For instance, IMS-088 had a striking impact on neurofilament disorganization in mice infused with ALS-CSF. The ALS-CSF exposure led to substantial reduction in protein levels for NfL, NfM, and NfH (Figure 2K–M) and especially to important cytoplasmic mislocalization of NfL (Figure 2J). It is now well established that neurofilament disorganization can cause axonal transport defects, neuronal dysfunction, and synaptic defects [35,36,37,38]. Here, the decreased levels of NF proteins in ALS-CSF-infused mice might be caused in part by translational suppression of protein synthesis due to excess cytoplasmic TDP-43 binding to 3′-UTR of NF mRNAs [14]. The IMS-088 treatment was able to prevent the abnormal cytoplasmic NfL accumulations in spinal neurons induced by i.c.v. infusion of ALS-CSF. IMS-088 was effective in preventing loss of NfL distribution in neurites but it did not restore the total NfL levels, which is likely reflecting a neuronal loss which perhaps may have occurred in the two days preceding the initiation of drug treatment. IMS-088 was effective in mitigating TDP-43 proteinopathy and formation of peripherin aggregates in mice infused with CSF from sporadic ALS (Figure 3). Both IMS-088 and Withaferin-A were reported to alleviate TDP-43 pathology in a mouse model of familial ALS-FTD expressing mutant TDP-43 [10]. IMS-088 also alleviated disease in mice expressing mutant FUSR521G [15]. Inhibition of NF-κB, a transcription factor that regulates cellular expression of many pro-inflammatory factors, by the root extract of the medicinal herbal plant *Withania somnifera,* mitigated TDP-43 proteinopathy and improved motor performance of a transgenic mouse model of ALS/FTLD with TDP-43 pathology [23]. Withaferin-A has been reported to extend the survival of SOD1 mutant ALS mice [11] and improved cognitive functions, induced autophagy, and attenuated TDP-43 proteinopathy in motor neurons through NF-κB inhibition, in various mice models of ALS [10,11,39].

Non-cell autonomous propagation, glial activation, and neuroinflammation are prominent modes of exacerbation of ALS-CSF mediated neuronal pathology [8,25,40]. Many studies have reported the anti-inflammatory effects of *Withania somnifera* extracts as well as Withaferin-A, the parent compound of IMS-088, in different mice models [10,11,23]. Our study provides evidence for the anti-inflammatory role of IMS-088 in mitigating astroglial and microglial activation. Morphological changes in microglia are linked to neuronal function and reflect altered physiology with a diverse and widespread impact on neuronal physiology [24]. While the microglial phenotype in the ALS-CSF group can be attributed to the neuroinflammation evident in the present model [8], the distinct morphology in response to IMS-088 treatment could reflect reparative adaptations, in line with the neuroprotective effects of IMS-088 observed in the current study. IMS-088 also prevented the increased expression levels of the enzymatically active ALS-CSF biomarker Chit1 (~39 kDa) [41]. These findings, coupled with the reduced expression and count of microglia expressing activation marker CD68, support the view of a post-inflammatory/restorative phase resulting from the IMS-088 treatment. Protective effects of active components of *Withania somnifera* have been reported to extend beyond inflammation. A study reported neuroprotection conferred by Withanone pre-treatment on reactive oxygen species (ROS), mitochondrial pathology, apoptosis, and DNA damage in Neuro2A cells exposed to NMDA excitotoxicity [42]. In the present study, too, the major neuronal processes altered at the translational level post-IMS-088 treatment of ALS-CSF-infused mice included mitochondrial pathology, altered energy metabolism, and apoptosis (Figure 5). Several studies and reviews have discussed the relevance of energy metabolism and mitochondrial integrity in ALS pathology, and its link to neuronal apoptosis [8,43,44,45,46]. These studies emphasized the importance of targeting metabolic dysfunction as well as mitochondrial pathology for therapeutic interventions. Here, our study adds important evidence for metabolic pathways being a putative target for treatment, while also elucidating the therapeutic potential of the novel drug IMS-088. Noteworthy here is the role of PGC1α, the transcriptional coactivator that regulates mitochondrial homeostasis and turnover by maintaining a balance between mitophagy and mitogenesis [28,47,48]. We observed its upregulation in spinal cords in response to ALS-CSF infusion, which was dramatically enhanced further upon IMS-088 treatment. PGC1α has been reported to influence the expression of not only mitochondrial genes but also of NFs affected in the present study, namely NfM and NfH, and it favors neuroprotective phenotypes in microglia [47,49]. Moreover, PGC1α positively modulates metabolism, physiology, and denervation-induced mitophagy in skeletal muscles, as well as remodeling of NMJs by enhancing NMJ size and integrity, also observed in response to IMS-088 in the present study [33,48].

Our proteomic results revealed the involvement of three major cellular and extracellular waste removal/clearance pathways in IMS-088-mediated neuroprotection, namely UBN-mediated degradation/Ubiquitin–Proteasome System (UPS), the phagosomal pathway, and the endosome-mediated cross-presentation of soluble exogenous antigens (Figure 5E). UPS has extensively been considered as a potential therapeutic target for neurodegenerative disorders owing to its important role in degrading intracellular proteins [50]. In achieving this, the pathway works closely with autophagy–lysosome pathways, and SQSTM1 acts as a central regulator in linking these two processes [29,51,52]. Accumulation of SQSTM1 is linked to inhibition of autophagy, which then leads to perturbation in sequestration of the ubiquitinated proteins for proteasomal degradation, as well as early neuromuscular denervation of the skeletal muscles (tibialis anterior), as also observed in the present study [52,53].

Ubiquitinated TDP-43 is a hallmark of ALS and the inability of the dysfunctional UPS to clear cytotoxic TDP-43 aggregates results in increased cytoplasmic TDP-43 aggregates causing the formation of stress granules and further neuronal damage [reviewed by [20]]. Accumulated SQTSM1 observed in our study (Figure 6C,D) could be sequestered into TDP-43 aggregates as reported in ALS patients, resulting in UPS dysfunction as well as the inhibition autophagy, further promoting misfolding and aggregation [54]. Furthermore, peripherin aggregates seen in our study (Figure 3A) are commonly observed in Bunina bodies which have been reported to coexist and/or colocalize with TDP-43 inclusions and are degraded through autophagy [55,56,57]. We also observed an effect of IMS-088 on the antigen-presenting endosomal pathways, which has also been reported to be regulated by autophagy [58,59]. In this role, SQSTM1 acts as a signaling mediator in late endosome and lysosome that targets ubiquitinated proteins for entry into autophagosomes. Lastly, SQSTM1 also regulates mitochondrial physiology through mitophagy with relevance to neurodegeneration [51]. These results highlight for the first time the closely coordinated impact of UPS and autophagy, namely the “autophago-proteasome”, in ALS pathology as well as the potential of IMS-088 to attenuate collective dysfunction [60].

One of the limitations of our study is the IMS-088 treatment regime that was initiated 48 h after the ALS-CSF infusion. This pilot study aimed to establish the relevance of the drug for treatment in the already diseased mice. Consequently, future studies need to be conducted with IMS-088 pre-treatment, to ascertain whether the partial protection conferred by the drug is a result of limited potential of the drug or because of irreversible neurogenerative events occurring within the first 48 hrs of i.c.v. infusion of ALS-CSF.

In conclusion, there were robust protective effects in mice infused with ALS-CSF of inhibiting NF-κB with oral administration of a novel Withaferin-A derivative called IMS-088. Our study highlights positive drug effects on motor performance, muscle pathology and innervation, motor neuron viability, and neuroinflammation. Of particular interest was the finding that IMS-088 administration reduced TDP-43 pathology and neurofilament disorganization, which are hallmarks of ALS. The proteomic analyses of immunoprecipitated neuronal ribosomes revealed the effects of IMS-088 on multiple pathways related to cytoskeletal changes, inflammation, metabolic dysfunction, mitochondria, UPS, and autophagy dysfunction. The translatome profiles suggest that perhaps combinatorial drug approaches should be considered for future treatments of ALS. Yet, the protective effects of IMS-088 in a mouse model based on infusion of CSF from sporadic ALS patients suggest that the NF-κB signaling pathway represents a compelling therapeutic target for sporadic ALS cases.

## Figures and Tables

**Table 1:** List of the antibodies.

Antibody	Species	Company	#Cat.	Dilution		Temp	h
				IF	WB		
Anti-Caspase-3	Rabbit polyclonal	Cell signaling technology, Danvers, MA, USA	#9661	-	1:1000	4 °C	24
Anti-CD68	Rat monoclonal	Biorad, Mississauga, ON, Canada		1:500	1:1000	4 °C	24
Anti-ChAT	Rabbit polyclonal	MilliporeSigma, Oakville, ON, Canada	AB143	-	1:1000	4 °C	24
Anti-Chit1	Rabbit polyclonal	SCBT, Dallas, TX, USA	SC46853	1:500	1:1000	4 °C	24
Anti-GAPDH	Mouse monoclonal	SCBT	SC32233	-	1:2000	4 °C	24
Anti-GFAP	Mouse monoclonal	Cell signaling Technology	#3670	1:800	1:2000	4 °C	24
Anti-Mitofilin	Rabbit polyclonal	Invitrogen ThermoFisher, Waltham, MA, USA	PA3870	1:500	-	4 °C	24
Anti-NfL	Rabbit polyclonal	MilliporeSigma	AB5294	1:500	1:1000	4 °C	24
Anti-NfM	Rabbit polyclonal	MilliporeSigma	AB1981	1:500	1:1000	4 °C	24
Anti-NfH	Mouse monoclonal	Abcam, Toronto, ON, Canada	AB7796	1:500	1:1000	4 °C	24
Anti-NeuN	Mouse monoclonal	MilliporeSigma	AB377	1:500	-	4 °C	24
Anti-Peripherin	Mouse monoclonal	MilliporeSigma	AB1527	1:500	-	4 °C	24
Anti-PGC1α	Mouse monoclonal	SCBT	SC-517380	1:500	-	4 °C	24
Anti-SQSTM1	Rabbit monoclonal	Cell signaling Technology	#5114	1:500	1:1000	4 °C	24
Anti-TDP-43	Rabbit polyclonal	Proteintech, Rosemont, IL, USA	#10782	1:800	1:5000	4 °C	24
Anti-TDP-43 (pSer 409/410)	Rabbit polyclonal	Cosmo Bio, Carlsbad, CA, USA	TIP-PTD-P01	-	1:1000	4 °C	24
Anti-UBN	Mouse monoclonal	MilliporeSigma	AB1510	1:500	1:1000	4 °C	24
Anti-Mouse IgG-HRP	Goat polyclonal	Invitrogen ThermoFisher	G-21040	-	1:5000	20 °C	1
Anti-mouse IgG-IRdye-680	Goat polyclonal	Li-Cor Biosciences, Lincoln, NE, USA	#926-8070	-	1:10,000	20 °C	1
Anti-rabbit IgG-Alexa 488	Goat polyclonal	Invitrogen ThermoFisher	#A32731	1:500	-	20 °C	1
Anti-rabbit IgG-IRdye-800	Goat polyclonal	Li-Cor Biosciences	#926-32211	-	1:10,000	20 °C	1
Anti-rabbit IgG-Alexa 568	Goat polyclonal	Invitrogen ThermoFisher	#A-11036	1:500	-	20 °C	1
Bungarotoxin-Alexa594		Invitrogen Thermofischer	B13423	1:500	-	4 °C	24

## Data Availability

Data are available on reasonable request, from the first and corresponding authors.

## References

[1] Cady J., Allred P., Bali T., Pestronk A., Goate A., Miller T.M., Mitra R.D., Ravits J., Harms M.B., Baloh R.H. (2015). Amyotrophic lateral sclerosis onset is influenced by the burden of rare variants in known amyotrophic lateral sclerosis genes. Ann. Neurol..

[2] Kunst C.B. (2004). Complex genetics of amyotrophic lateral sclerosis. Am. J. Hum. Genet..

[3] Turner M.R., Al-Chalabi A., Chio A., Hardiman O., Kiernan M.C., Rohrer J.D., Rowe J., Seeley W., Talbot K. (2017). Genetic screening in sporadic ALS and FTD. J. Neurol. Neurosurg. Psychiatry.

[4] van Es M.A., Hardiman O., Chio A., Al-Chalabi A., Pasterkamp R.J., Veldink J.H., van den Berg L.H. (2017). Amyotrophic lateral sclerosis. Lancet.

[5] Iguchi Y., Eid L., Parent M., Soucy G., Bareil C., Riku Y., Kawai K., Takagi S., Yoshida M., Katsuno M. (2016). Exosome secretion is a key pathway for clearance of pathological TDP-43. Brain J. Neurol..

[6] Mitchell J.C., Constable R., So E., Vance C., Scotter E., Glover L., Hortobagyi T., Arnold E.S., Ling S.C., McAlonis M. (2015). Wild type human TDP-43 potentiates ALS-linked mutant TDP-43 driven progressive motor and cortical neuron degeneration with pathological features of ALS. Acta Neuropathol. Commun..

[7] Smith R., Myers K., Ravits J., Bowser R. (2015). Amyotrophic lateral sclerosis: Is the spinal fluid pathway involved in seeding and spread?. Med. Hypotheses.

[8] Mishra P.S., Boutej H., Soucy G., Bareil C., Kumar S., Picher-Martel V., Dupre N., Kriz J., Julien J.P. (2020). Transmission of ALS pathogenesis by the cerebrospinal fluid. Acta Neuropathol. Commun..

[9] Zhou Z., Xiang W., Jiang Y., Tian N., Wei Z., Wen X., Wang W., Liao W., Xia X., Li Q. (2020). Withaferin A alleviates traumatic brain injury induced secondary brain injury via suppressing apoptosis in endothelia cells and modulating activation in the microglia. Eur. J. Pharmacol..

[10] Kumar S., Phaneuf D., Julien J.P. (2021). Withaferin-A Treatment Alleviates TAR DNA-Binding Protein-43 Pathology and Improves Cognitive Function in a Mouse Model of FTLD. Neurotherapeutics.

[11] Patel P., Julien J.P., Kriz J. (2015). Early-stage treatment with Withaferin A reduces levels of misfolded superoxide dismutase 1 and extends lifespan in a mouse model of amyotrophic lateral sclerosis. Neurotherapeutics.

[12] Jackson S.S., Oberley C., Hooper C.P., Grindle K., Wuerzberger-Davis S., Wolff J., McCool K., Rui L., Miyamoto S. (2015). Withaferin A disrupts ubiquitin-based NEMO reorganization induced by canonical NF-κB signaling. Exp. Cell Res..

[13] Dutta K., Thammisetty S.S., Boutej H., Bareil C., Julien J.P. (2020). Mitigation of ALS Pathology by Neuron-Specific Inhibition of Nuclear Factor Kappa B Signaling. J. Neurosci..

[14] Kumar S., Phaneuf D., Cordeau P., Boutej H., Kriz J., Julien J.P. (2021). Induction of autophagy mitigates TDP-43 pathology and translational repression of neurofilament mRNAs in mouse models of ALS/FTD. Mol. Neurodegener..

[15] Pelaez M.C., Desmeules A., Gelon P.A., Glasson B., Marcadet L., Rodgers A., Phaneuf D., Pozzi S., Dutchak P.A., Julien J.P. (2023). Neuronal dysfunction caused by FUSR521G promotes ALS-associated phenotypes that are attenuated by NF-κB inhibition. Acta Neuropathol. Commun..

[16] Pathan M., Keerthikumar S., Chisanga D., Alessandro R., Ang C.S., Askenase P., Batagov A.O., Benito-Martin A., Camussi G., Clayton A. (2017). A novel community driven software for functional enrichment analysis of extracellular vesicles data. J. Extracell. Vesicles.

[17] Xia J., Gill E.E., Hancock R.E. (2015). NetworkAnalyst for statistical, visual and network-based meta-analysis of gene expression data. Nat. Protoc..

[18] Hepple R.T. (2018). When motor unit expansion in ageing muscle fails, atrophy ensues. J. Physiol..

[19] Lomen-Hoerth C., Anderson T., Miller B. (2002). The overlap of amyotrophic lateral sclerosis and frontotemporal dementia. Neurology.

[20] Suk T.R., Rousseaux M.W.C. (2020). The role of TDP-43 mislocalization in amyotrophic lateral sclerosis. Mol. Neurodegener..

[21] Ratti A., Buratti E. (2016). Physiological functions and pathobiology of TDP-43 and FUS/TLS proteins. J. Neurochem..

[22] Gomez-Pinedo U., Galan L., Yanez M., Matias-Guiu J., Valencia C., Guerrero-Sola A., Lopez-Sosa F., Brin J.R., Benito-Martin M.S., Leon-Espinosa G. (2018). Histological changes in the rat brain and spinal cord following prolonged intracerebroventricular infusion of cerebrospinal fluid from amyotrophic lateral sclerosis patients are similar to those caused by the disease. Neurologia.

[23] Dutta K., Patel P., Rahimian R., Phaneuf D., Julien J.P. (2017). Withania somnifera Reverses Transactive Response DNA Binding Protein 43 Proteinopathy in a Mouse Model of Amyotrophic Lateral Sclerosis/Frontotemporal Lobar Degeneration. Neurotherapeutics.

[24] Morrison H., Young K., Qureshi M., Rowe R.K., Lifshitz J. (2017). Quantitative microglia analyses reveal diverse morphologic responses in the rat cortex after diffuse brain injury. Sci. Rep..

[25] Varghese A.M., Sharma A., Mishra P., Vijayalakshmi K., Harsha H.C., Sathyaprabha T.N., Bharath S.M., Nalini A., Alladi P.A., Raju T.R. (2013). Chitotriosidase—A putative biomarker for sporadic amyotrophic lateral sclerosis. Clin. Proteom..

[26] Renkema G.H., Boot R.G., Strijland A., Donker-Koopman W.E., van den Berg M., Muijsers A.O., Aerts J.M. (1997). Synthesis, sorting, and processing into distinct isoforms of human macrophage chitotriosidase. Eur. J. Biochem..

[27] Blasiak J., Pawlowska E., Szczepanska J., Kaarniranta K. (2019). Interplay between autophagy and the ubiquitin-proteasome system and its role in the pathogenesis of age-related macular degeneration. Int. J. Mol. Sci..

[28] Summermatter S., Santos G., Perez-Schindler J., Handschin C. (2013). Skeletal muscle PGC-1alpha controls whole-body lactate homeostasis through estrogen-related receptor alpha-dependent activation of LDH B and repression of LDH A. Proc. Natl. Acad. Sci. USA.

[29] Seibenhener M.L., Babu J.R., Geetha T., Wong H.C., Krishna N.R., Wooten M.W. (2004). Sequestosome 1/p62 Is a Polyubiquitin Chain Binding Protein Involved in Ubiquitin Proteasome Degradation. Mol. Cell. Biol..

[30] Keller B.A., Volkening K., Droppelmann C.A., Ang L.C., Rademakers R., Strong M.J. (2012). Co-aggregation of RNA binding proteins in ALS spinal motor neurons: Evidence of a common pathogenic mechanism. Acta Neuropathol..

[31] Wong J.K., Roselle A.K., Shue T.M., Shimshak S.J.E., Beaty J.M., Celestin N.M., Gao I., Griffin R.P., Cudkowicz M.E., Sadiq S.A. (2022). Apolipoprotein B-100-mediated motor neuron degeneration in sporadic amyotrophic lateral sclerosis. Brain Commun..

[32] Nishimune H., Stanford J.A., Mori Y. (2014). Role of exercise in maintaining the integrity of the neuromuscular junction. Muscle Nerve.

[33] Arnold A.S., Gill J., Christe M., Ruiz R., McGuirk S., St-Pierre J., Tabares L., Handschin C. (2014). Morphological and functional remodelling of the neuromuscular junction by skeletal muscle PGC-1alpha. Nat. Commun..

[34] Lee D.H., Ahn J., Jang Y.J., Seo H.D., Ha T.Y., Kim M.J., Huh Y.H., Jung C.H. (2020). Withania somnifera Extract Enhances Energy Expenditure via Improving Mitochondrial Function in Adipose Tissue and Skeletal Muscle. Nutrients.

[35] Lee M.K., Marszalek J.R., Cleveland D.W. (1994). A mutant neurofilament subunit causes massive, selective motor neuron death: Implications for the pathogenesis of human motor neuron disease. Neuron.

[36] Cote F., Collard J.F., Julien J.P. (1993). Progressive neuronopathy in transgenic mice expressing the human neurofilament heavy gene: A mouse model of amyotrophic lateral sclerosis. Cell.

[37] Wong N.K., He B.P., Strong M.J. (2000). Characterization of neuronal intermediate filament protein expression in cervical spinal motor neurons in sporadic amyotrophic lateral sclerosis (ALS). J. Neuropathol. Exp. Neurol..

[38] Didonna A., Opal P. (2019). The role of neurofilament aggregation in neurodegeneration: Lessons from rare inherited neurological disorders. Mol. Neurodegener..

[39] Swarup V., Phaneuf D., Dupré N., Petri S., Strong M., Kriz J., Julien J.-P. (2011). Deregulation of TDP-43 in amyotrophic lateral sclerosis triggers nuclear factor κB-mediated pathogenic pathways. J. Exp. Med..

[40] Mishra P.S., Vijayalakshmi K., Nalini A., Sathyaprabha T.N., Kramer B.W., Alladi P.A., Raju T.R. (2017). Etiogenic factors present in the cerebrospinal fluid from amyotrophic lateral sclerosis patients induce predominantly pro-inflammatory responses in microglia. J. Neuroinflamm..

[41] Steinacker P., Verde F., Fang L., Feneberg E., Oeckl P., Roeber S., Anderl-Straub S., Danek A., Diehl-Schmid J., Fassbender K. (2018). Chitotriosidase (CHIT1) is increased in microglia and macrophages in spinal cord of amyotrophic lateral sclerosis and cerebrospinal fluid levels correlate with disease severity and progression. J. Neurol. Neurosurg. Psychiatry.

[42] Dar N.J., Bhat J.A., Satti N.K., Sharma P.R., Hamid A., Ahmad M. (2017). Withanone, an Active Constituent from Withania somnifera, Affords Protection Against NMDA-Induced Excitotoxicity in Neuron-Like Cells. Mol. Neurobiol..

[43] Vandoorne T., De Bock K., Van Den Bosch L. (2018). Energy metabolism in ALS: An underappreciated opportunity?. Acta Neuropathol..

[44] Bolanos J.P., Moro M.A., Lizasoain I., Almeida A. (2009). Mitochondria and reactive oxygen and nitrogen species in neurological disorders and stroke: Therapeutic implications. Adv. Drug Deliv. Rev..

[45] Flachbartova Z., Kovacech B. (2013). Mortalin—A multipotent chaperone regulating cellular processes ranging from viral infection to neurodegeneration. Acta Virol..

[46] Sharma A., Varghese A.M., Vijaylakshmi K., Sumitha R., Prasanna V.K., Shruthi S., Chandrasekhar Sagar B.K., Datta K.K., Gowda H., Nalini A. (2016). Cerebrospinal Fluid from Sporadic Amyotrophic Lateral Sclerosis Patients Induces Mitochondrial and Lysosomal Dysfunction. Neurochem. Res..

[47] Lin J., Wu P.H., Tarr P.T., Lindenberg K.S., St-Pierre J., Zhang C.Y., Mootha V.K., Jager S., Vianna C.R., Reznick R.M. (2004). Defects in adaptive energy metabolism with CNS-linked hyperactivity in PGC-1alpha null mice. Cell.

[48] Vainshtein A., Desjardins E.M., Armani A., Sandri M., Hood D.A. (2015). PGC-1alpha modulates denervation-induced mitophagy in skeletal muscle. Skelet. Muscle.

[49] Mou C., Liu B., Wang M., Jiang M., Han T. (2014). PGC-1-Related Coactivator (PRC) Is an Important Regulator of Microglia M2 Polarization. J. Mol. Neurosci..

[50] Gong B., Radulovic M., Figueiredo-Pereira M.E., Cardozo C. (2016). The Ubiquitin-Proteasome System: Potential Therapeutic Targets for Alzheimer’s Disease and Spinal Cord Injury. Front. Mol. Neurosci..

[51] Shin W.H., Park J.H., Chung K.C. (2020). The central regulator p62 between ubiquitin proteasome system and autophagy and its role in the mitophagy and Parkinson’s disease. BMB Rep..

[52] Korolchuk V.I., Mansilla A., Menzies F.M., Rubinsztein D.C. (2009). Autophagy Inhibition Compromises Degradation of Ubiquitin-Proteasome Pathway Substrates. Mol. Cell.

[53] Rudnick N.D., Griffey C.J., Guarnieri P., Gerbino V., Wang X., Piersaint J.A., Tapia J.C., Rich M.M., Maniatis T. (2017). Distinct roles for motor neuron autophagy early and late in the SOD1(G93A) mouse model of ALS. Proc. Natl. Acad. Sci. USA.

[54] Brady O.A., Meng P., Zheng Y., Mao Y., Hu F. (2011). Regulation of TDP-43 aggregation by phosphorylation andp62/SQSTM1. J. Neurochem..

[55] Mori F., Kakita A., Takahashi H., Wakabayashi K. (2014). Co-localization of Bunina bodies and TDP-43 inclusions in lower motor neurons in amyotrophic lateral sclerosis. Neuropathology.

[56] Mizuno Y., Fujita Y., Takatama M., Okamoto K. (2011). Peripherin partially localizes in Bunina bodies in amyotrophic lateral sclerosis. J. Neurol. Sci..

[57] Mori F., Miki Y., Kon T., Tanji K., Wakabayashi K. (2019). Autophagy Is a Common Degradation Pathway for Bunina Bodies and TDP-43 Inclusions in Amyotrophic Lateral Sclerosis. J. Neuropathol. Exp. Neurol..

[58] Fraser J., Simpson J., Fontana R., Kishi-Itakura C., Ktistakis N.T., Gammoh N. (2019). Targeting of early endosomes by autophagy facilitates EGFR recycling and signalling. EMBO Rep..

[59] Fraser J., Cabodevilla A.G., Simpson J., Gammoh N. (2017). Interplay of autophagy, receptor tyrosine kinase signalling and endocytic trafficking. Essays Biochem..

[60] Lenzi P., Lazzeri G., Biagioni F., Busceti C.L., Gambardella S., Salvetti A., Fornai F. (2016). The Autophagoproteasome a Novel Cell Clearing Organelle in Baseline and Stimulated Conditions. Front. Neuroanat..

